# Expressed *Vomeronasal Type-1 Receptors* (*V1rs*) in Bats Uncover Conserved Sequences Underlying Social Chemical Signaling

**DOI:** 10.1093/gbe/evz179

**Published:** 2019-08-19

**Authors:** Laurel R Yohe, Kalina T J Davies, Stephen J Rossiter, Liliana M Dávalos

**Affiliations:** 1 Department of Ecology and Evolution, Stony Brook University, NY; 2 Department of Geology & Geophysics, Yale University, New Haven, CT; 3 School of Biological and Chemical Sciences, Queen Mary University of London, United Kingdom; 4 Consortium for Inter-Disciplinary Environmental Research, Stony Brook University, Stony Brook, NY

**Keywords:** *V1r*, vomeronasal system, pheromone, gene tree, Chiroptera, chemosensation

## Abstract

In mammals, social and reproductive behaviors are mediated by chemical cues encoded by hyperdiverse families of receptors expressed in the vomeronasal organ. Between species, the number of intact receptors can vary by orders of magnitude. However, the evolutionary processes behind variation in receptor number, and its link to fitness-related behaviors are not well understood. From vomeronasal transcriptomes, we discovered the first evidence of intact *vomeronasal type-1 receptor* (*V1r*) genes in bats, and we tested whether putatively functional bat receptors were orthologous to those of related taxa, or whether bats have evolved novel receptors. Instead of lineage-specific duplications, we found that bat *V1r*s show high levels of orthology to those of their relatives, and receptors are under comparative levels of purifying selection as non-bats. Despite widespread vomeronasal organ loss in bats, *V1r* copies have been retained for >65 million years. The highly conserved nature of bat *V1r*s challenges our current understanding of mammalian *V1r* function and suggests roles other than conspecific recognition or mating initiation in social behavior.

Nearly all mammals can perceive pheromones—broadly construed as any olfactory cue excreted from individuals of a different species or conspecific (Silva and Antunes 2017)—though there is great variation in the genetic detection mechanisms and morphological structures involved (Meisami and Bhatnagar 1998; Grus et al. 2007; Young et al. 2010). Mammalian pheromone detection, or vomerolfaction (Cooper and Burghardt 1992), mediates key social and reproductive behaviors including mating and courtship, parental care, conspecific identification, and territoriality (Liberles 2014). Pheromone detection occurs in the vomeronasal organ, a structure composed of a cluster of sensory neurons in the nasal anterior that expresses ultrasensitive G-protein coupled receptors [e.g. vomeronasal type-1 receptors (V1Rs), vomeronasal type-2 receptors (V2Rs)]. These receptors bind to pheromones (Ibarra-Soria et al. 2014), and trigger a signaling cascade that activates the Transient receptor potential cation channel 2 (Trpc2) ion channel resulting in depolarization, so the cue can be processed by the brain (Mast et al. 2010). However, pinpointing which of the hundreds of receptors mediates a given behavior is challenging. A comparative approach can narrow down the scope of functional characterization, as understanding the gene history and molecular evolution across divergent lineages can help determine which receptors are relevant to particular species. Here, we analyze the diversity of mammalian *V1r*s—focusing particularly on bats—and infer the processes responsible for their evolutionary history. We concentrate primarily on *V1r*s, as they show the greatest variation in number of genes across species of any mammalian gene family (Grus et al. 2007; Young et al. 2010), and dominate among vomeronasal receptors in placental mammals (Silva and Antunes 2017).

Because of its relevance in fitness-related behaviors, the vomeronasal organ is highly conserved across mammals; in most the morphology is well developed, the signaling pathway is maintained, and the receptors have diversified across species. There are, however, a few exceptional cases of vestigialization of vomerolfaction during mammalian diversification including several aquatic mammals, catarrhine primates, and many bats (Bhatnagar and Meisami 1998; Zhang and Webb 2003; Yu et al. 2010; Yohe et al. 2017). The relaxation of selection in these lineages has led to pseudogenization of many elements of the molecular pathways involved in pheromone detection and transduction (Zhang and Webb 2003; Young et al. 2010; Yu et al. 2010; Zhao et al. 2011; Yohe et al. 2017), and losses may be related to shifts to underwater or diurnal niches. No explanation has emerged, however, for variation in the maintenance of the vomeronasal system in bats, as more than a dozen independent functional losses in *Trpc2* gene function seem unrelated to either the evolution of flight, or of other specialized senses (Yohe et al. 2017).

*V1r*s play a role in species-specific behaviors (Grus and Zhang 2004; Ibarra-Soria et al. 2014), and may even play a role in speciation. For example, in rodents, orthologous receptors vary among species and subspecies, with less than 20% of genes shared between mouse and rat (Zhang et al. 2007; Park et al. 2011; Wynn et al. 2012). Duplications of *V1r*s prior to the diversification of lemurs and lorises expanded the number of intact *V1r*s by an order of magnitude (Yoder et al. 2014; Yoder and Larsen 2014), perhaps promoting strepsirrhine diversification as they colonized Madagascar. Like other chemosensory genes, *V1r*s evolve via a birth–death process by which gene copies frequently duplicate and pseudogenize over time (Nei and Rooney 2005). This birth–death process generates great variance in receptor numbers across species; for example, there are well over 200 *V1r*s in the platypus and mouse lemurs, fewer than 10 intact *V1r*s in catarrhine primates, and none were detected in either the bottlenose dolphin or the two species of bats previously analyzed (Young et al. 2010). Attempts to explain this variance have linked *V1r* numbers to nocturnality (Wang et al. 2010), but correlating numbers of receptors to functional ecology fails to address the evolutionary history of *V1r* repertoires. Here, we trace the phylogenetic history of each bat *V1r* gene and infer its orthology to determine whether each *V1r* is shared among divergent mammals, or instead is unique to a species or clade. Because *V1r*s have been shown to mediate species-specific behaviors that may be related to species boundaries, we hypothesized that bat *V1r*s have evolved through lineage-specific duplications and perhaps served as a key innovation that facilitated speciation of the Neotropical leaf-nosed bats (Phyllostomidae)—a species rich clade with diverse dietary adaptations and a conserved and intact *Trpc2* (Yohe et al. 2017). Alternatively, *V1r*s may be conserved orthologs of non-bat lineages. As orthologous chemosensory genes of divergent species will have a higher probability of detecting a similar compound than paralogs within a species (Adipietro et al. 2012), shared orthology among bats and non-bats could indicate that the receptor binds to similar ligands or mediates similar behaviors.

To test our hypotheses, we generated new transcriptomes from the vomeronasal organs of nine species of phyllostomids (supplementary table S1, Supplementary Material online), and we combined these with data from published genomes of 16 additional species (14 bats and 2 outgroups). Our data revealed at least one intact *V1r* in each transcriptome, thus providing the first evidence of transcribed *V1r*s in bats. The vampire bat (*Desmodus rotundus*) had 10 distinct expressed *V1r*s, the most of any of the bat species we examined (fig. 1). We validated these receptor transcripts with the *V1r* sequences identified from the recently published vampire bat genome (Zepeda Mendoza et al. 2018). All transcribed *V1r*s were found among the 14 intact *V1r* sequences identified in the genome (supplementary fig. S1, Supplementary Material online).

**Fig. 1.**
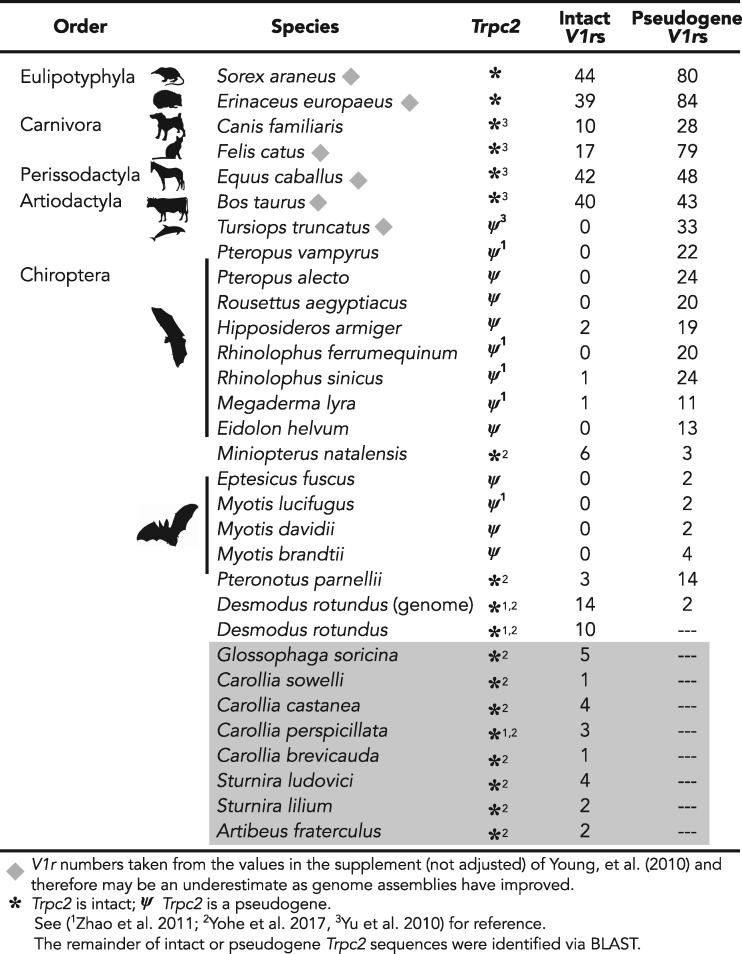
—Number of intact and pseudogenized *V1r*s among some laurasiatherians. *V1r*s from the transcriptomes are highlighted in gray. The remaining species were characterized from available genomes or are reported from Young et al. (2010). Pseudogenized *V1r*s are receptor genes with a frameshift or premature stop codon but with at least 650 bp. Vertical lines are bats that likely have a vestigial vomeronasal system, either based on morphology or *Trpc2*. Silhouettes are not to scale and were obtained from PhyloPic.

We also characterized intact and pseudogenized *V1r*s from all other available bat genomes, as well as from the horse and the dog, two outgroup representatives within Laurasiatheria. We identified very few, if any, intact *V1r*s in any other bat genome. An abundance of pseudogenized receptor genes were found in the exclusively Old World suborder Yinpterochiroptera, all of which also had pseudogenized *Trpc2* genes (fig. 1). Three species of yinpterochiropterans, of the 11 species predicted to lack a vomeronasal organ based on *Trpc2*, were an exception, with 1 to 2 *V1r*s possessing intact reading frames identified (fig. 1). Several intact *V1r*s were detected in *Miniopterus natalensis* and *Pteronotus cf. parnellii*, representatives of two non-phyllostomid families previously shown to have an intact *Trpc2* gene and intact vomeronasal organ (fig. 1). We also detected differences in receptors (between 3 and 6 genes) from the horse and dog genomes than had been previously reported (Young et al. 2010). Differences are likely due to the availability of newer versions of the dog and horse genomes and our use of hidden Markov models rather than BLAST to characterize *V1r*s. We emphasize, however, that the reported number of *V1r* genes per species should be considered a dynamic value and may change as genome assemblies and annotation methods improve.

To determine orthologous gene groups (orthogroups) of *V1r*s, we reconstructed unrooted trees and identified genes forming monophyletic groups across different species (Ballesteros and Hormiga 2016). We pruned the gene tree into orthogroups while also allowing in-paralogs, genes within an orthogroup duplicated because a species diverged, to remain in the tree. Eighteen orthogroups were recovered, but five of these orthogroups contained only a single gene and many contained only two or three genes. Thus, we recovered a total of three orthogroups (fig. 2*A*–*C*), and two of these contained enough sequence data for subsequent analyses of molecular evolution (fig. 2*A* and *C*). There were no orthogroups with more than six genes that solely contained bats, suggesting all bats share orthologs with either the horse or dog lineage.

**Fig. 2.**
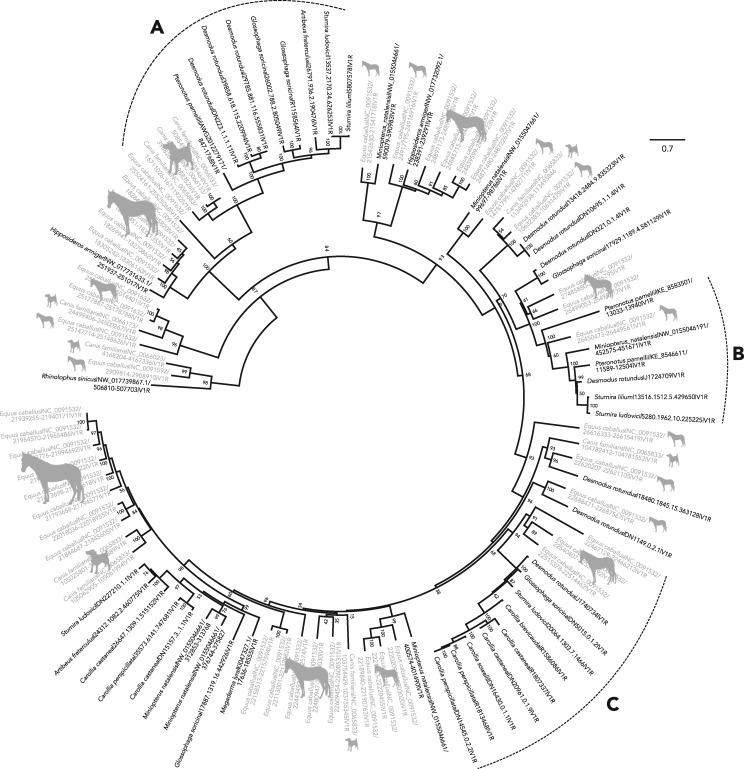
—Codon model gene tree for intact *V1r*s identified from the vomeronasal organ transcriptomes of bats (black names), the few functional *V1r*s from bat genomes (also in black), and the genomes of *Equus caballus* and *Canis familiaris* (gray names). Node labels are bootstrap support values. Numbers on the tip label gene correspond to the unique transcriptome assembly number or the genome location for newly identified genomic sequences. Letter labels indicate orthogroups identified from the UPhO analysis that resulted in more than 5 taxa and included any non-bats. Silhouettes were obtained from PhyloPic.

In mice, lemurs, and marsupials, considerable variation in *V1r* copies among species suggests vomerolfaction mediates species recognition, and possibly speciation (Grus et al. 2005; Wynn et al. 2012; Yoder et al. 2014). Although most bats with transcribed *V1r*s are found within the recently radiated Neotropical leaf-nosed bats (Dumont et al. 2012), the small number of species-specific paralogs combined with the 100% orthology between bat receptors and those from the horse and dog (compared with ∼10% orthology seen in mouse and 16% in rats; Grus and Zhang 2004; Zhang et al. 2007; Ohara et al. 2009) together suggest that they play no role in species recognition. Hence, the low *V1r* diversity in bats implies an alternative function for these receptors. Comparisons in ruminants (cow, sheep, and goat) revealed conserved *V1r* repertoires with up to 70% orthology between species, but very little overlap with rodent *V1r* repertoires (Ohara et al. 2009). Like other laurasiatherians (Keller and Lévy 2012), bats display a high degree of orthology with their relatives. Such sequence conservation hints at function mediating innate behaviors common to all laurasiatherians, as the vomeronasal neurons that express *V1r*s are hardwired to a common region of the brain responsible for similar instinctive behaviors (Bear et al. 2016), including mating, predator detection, and parental care. Although the receptors may differ in the compounds they bind to as a result of amino acid differences among lineages; thus sequence conservation and orthology imply functions shared by all laurasiatherian species rather than species-specific roles.

To test for Darwinian selection in bat *V1r*s, we estimated the ratio of nonsynonymous to synonymous substitution rates (*ω*) for bats and compared them with the background rate including genes from the horse and dog. First considering rates for the entire tree of intact *V1r*s, we found no significant difference between rates in bats and other species [table 1 {PAML}: χ^2^_(1)_ = 2.1 *P* = 0.15; table 2 {RELAX}: χ^2^_(1)_ = 0.65; *P* = 0.42], suggesting similar evolutionary processes are shaping the *V1r* repertoires of bats and non-bats. This rejects our hypothesis that bats have evolved bat-specific receptors in response to diversification of phyllostomids. Nevertheless, rates of *V1r* molecular evolution are relatively high in both bats and their sampled relatives. Both across the entire phylogeny of intact receptors and within orthogroups (tables 1 and 2), there were at least 51%, and sometimes as many as 69%, of codon sites evolving neutrally (*ω* = 1.0) in both bats and non-bats. Chemosensory genes are among the fastest-evolving in the mammalian genome, second only to genes involved in pathogen-recognition (Wynn et al. 2012; Yoder and Larsen 2014). As the neural mechanisms of signal processing are highly conserved in vertebrates (Bear et al. 2016), the duplicative nature of these genes and the high rates of evolution likely reflect fine-tuning of the detection of ever-changing environmental chemical space.

**Table 1:** Results from the PAML Clade Models

Model	lnL	np	*κ*	TL	LR	*P*	*ω* Site Classes		
							*ω*_1_	*ω*_2_	*ω*_3_
Whole tree									
* M2a_rel (null)*	−47,415	198	2.29	51.0	—	—			
* ω*_background_							0.16 (12%)	1.0 (51%)	0.47 (37%)
* Clade Model C*	−47,414	199	2.29	51.0	2.1	0.15			
* ω*_background_							0.15 (12%)	1.0 (51%)	0.45 (37%)
* ω*_bats_							0.15 (12%)	1.0 (51%)	0.50 (37%)
Orthogroup A									
* M2a_rel (null)*	−5,446	29	2.64	4.90	—	—			
* ω*_background_							0.15 (39%)	1.0 (56%)	4.21 (6%)
* Clade Model C*	−5,456	30	2.48	4.59	20.3	6.6e-6			
* ω*_background_							0.13 (36%)	1.0 (56%)	1.02 (8%)
* ω*_bats_							0.13 (36%)	1.0 (56%)	0.00 (8%)
Orthogroup C									
* M2a_rel (null)*	−4,979	27	2.54	3.56	—	—			
* ω*_background_							0.00 (11%)	1.0 (57%)	0.35 (32%)
* Clade Model C*	−4,963	28	2.56	3.56	30.8	2.8e-8			
* ω*_background_							0.07 (21%)	1.0 (52%)	1.34 (27%)
* ω*_bats_							0.07 (21%)	1.0 (52%)	0.02 (27%)

Note.—The gray box indicates the selected model or the null model not rejected based on the likelihood ratio test. Values for the codon site classes are *ω* estimates for each of the three codon site classes: purifying (*ω*_1_), neutral (*ω*_2_), and varying (*ω*_3_). The percentages in parentheses are the proportion of codon sites found within that respective codon site class. Orthogroup B had too few species to have power for a Clade Model C analysis.

*κ*, transition/transversion rate; lnL, log-likelihood; LR, likelihood ratio; np, number of parameters; *P*, *P* value of likelihood ratio of alternative relative to null for each test; TL, tree length.

**Table 2:** Results from RELAX Analyses

Model	lnL	np	AIC_c_	*k*	LR	*P*	*ω* Site Classes		
							*ω*_1_	*ω*_2_	*ω*_3_
*Null*	−47,388	212	95,204	1	—	—			
					*ω*_background_		0.00 (39%)	1 (59%)	31.6 (2%)
					*ω*_bats_		0.00 (39%)	1 (59%)	31.6 (2%)
*Alternative*	−36,748	169	73,837	1.05	0.65	0.42			
					*ω*_background_		0.00 (39%)	1 (59%)	29.7 (2%)
					*ω*_bats_		0.00 (39%)	1 (59%)	35.5 (2%)

Note.—Values for the codon site classes are *ω* estimates for each of the three codon site classes: purifying (*ω*_1_), neutral (*ω*_2_), and varying (*ω*_3_). The percentage values in parentheses are the proportion of codon sites found within that respective codon site class. The gray box indicates the model with the best fit, demonstrating the lowest AICc.

AIC_c_, sample-sized corrected Akaike Information Criterion; *k*, selection intensity; lnL, log-likelihood; LR, likelihood ratio; np, number of parameters; *P*, *P* value of likelihood ratio of alternative relative to null for each test.

Contrary to what is seen in the gene tree as a whole, orthogroups may be evolving differently in bats and non-bats. For both *V1r* orthogroups, horses have a higher rate than bats (supplementary table S3, Supplementary Material online), indicating potential clade-specific adaptation of particular receptors. There were significant differences between bats and non-bats in all three orthogroups (table 1; A: χ^2^_(1)_ = 20.3 *P* = 6.6e-6; C: χ^2^_(1)_ = 30.8 *P* = 2.8e-8). For Orthogroup A, a few codon sites (8%) were evolving at a neutral rate in horses, and at a purifying rate in bats. In Orthogroup C, bats showed very low *ω* rates relative to the background branches for 27% of the codon sites, indicating strong purifying selection in bats for these genes but neutral or slight positive rates for horses.

Both putatively functional and pseudogenized bat *V1r*s illuminate the evolutionary processes shaping the vomeronasal system as a whole (Yohe and Dávalos 2018). The same copies of some receptors have been maintained because the ancestor of bats diverged from those of the horse or dog, as shown by both the high degree of orthology (fig. 2), and slight differences in rates of evolution between the intact receptors of bats and those of related non-bats. This finding bolsters the hypothesis that phyllostomid and miniopterid bats with seemingly intact vomeronasal systems retained function throughout bat diversification, whereas most other bat families independently lost function. Moreover, our data support the idea that all components of the vomeronasal system evolve together, resulting in an all-or-nothing pattern. Specifically, lineages with intact *V1R*s also have intact *Trpc2* (fig. 1) and well-developed vomeronasal organ morphology, whereas bat families with pseudogenized *Trpc2* and/or degraded morphology tend to lack intact receptors. Together with analyses correlating high rates of *Trpc2* codon substitutions and loss of the vomeronasal brain region (Yohe and Dávalos 2018), patterns of *V1r* pseudogenization in bats highlight the consequences of relaxed selection on molecular components of the system. Finally, the phylogeny of bat *V1r* pseudogenes also reveals that intact copies from the horse and dog are sometimes pseudogenized across all bats (fig. 3), even in species with intact *Trpc2*. Although these receptors are likely still relevant to the ecology of the horse and dog, their complete loss of function indicates they are no longer relevant to bats.

**Fig. 3.**
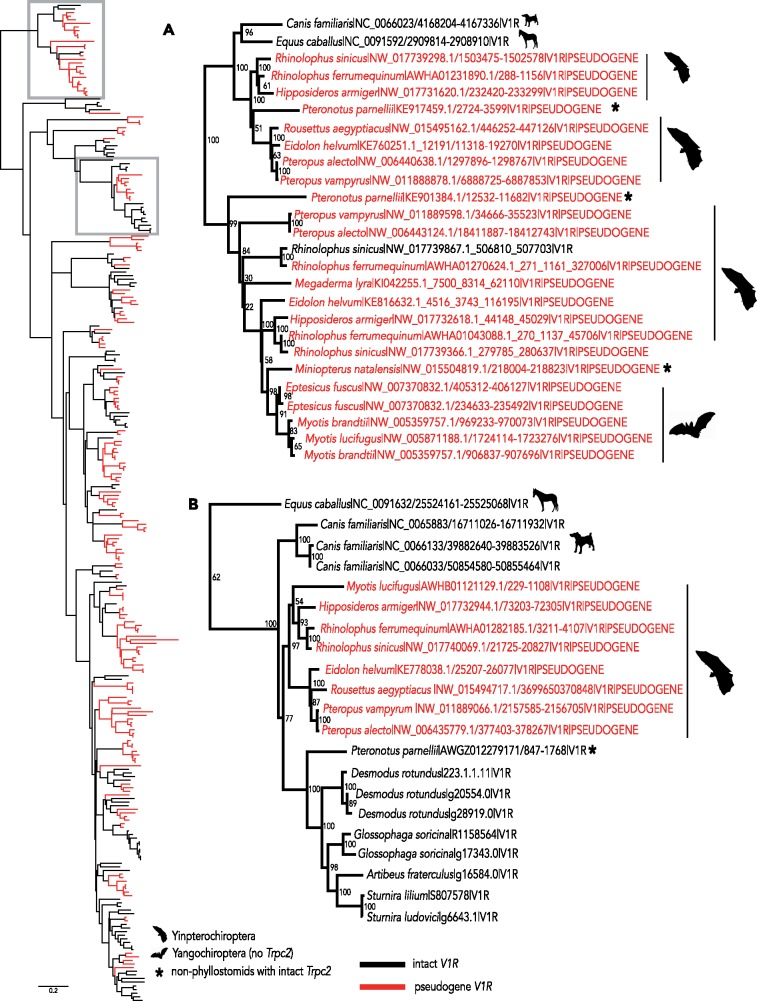
—Gene tree inferred under a nucleotide model evolution of intact *V1r*s from horse, dog, and bat, as well as pseudogenes identified from all bat genomes. Horse and dog pseudogenes were not included for clarity. Red branches and labels indicate pseudogenized genes and black indicates intact *V1r*s. Inset (*A*) shows a monophyletic group of genes in which the gene copy is intact in the ancestral dog, but has been lost in all bats, including species with an intact *Trpc2*. This orthogroup may be nonfunctional in phyllostomids, as there is no evidence it was expressed in the transcriptome. Inset (*B*) shows a monophyletic groups in which the gene copy is intact in the horse or dog, and most bats with intact *Trpc2*. However, the copy has been pseudogenized in yinpterochiropteran lineages, which lack an intact *Trpc2*.

Why some bats have completely lost vomeronasal function, while some have been under strong selection to retain it, remains a mystery. Receptors other than *V1r*s, such as those expressed in the main olfactory epithelium, may respond to pheromones becoming sufficient for detecting the relevant social chemical cues. Although *V2r*s, the other major vomeronasal receptor gene family, were not found in the transcriptomes of these bats, the dog and cow genomes also lack *V2r*s, and these genes might not be relevant in laurasiatherians (Grus et al. 2007). In fact, whereas pheromones are detected by vomeronasal receptors, vomeronasal receptors can detect other non-pheromone odorants and pheromones can be detected by other systems, such as the main olfactory system (Baxi et al. 2006; Isogai et al. 2011). In other words, the two chemosensory systems may not be mutually exclusive. In contrast to rodents, sheep and goats (also laurasiatherians) primarily use their main olfactory system for processing social chemical signals (Keller and Lévy 2012). The many genes expressed in the main olfactory epithelium, including the *major histocompatibility complex* (*MHC*), *trace amine-associated receptors* (*TAAR*s), and olfactory receptors, all have been shown to play a role in social chemical communication (Fortes-Marco et al. 2013; Li et al. 2013; López et al. 2014). An association between mate choice, and *MHC-*class 1 alleles and variation within *TAAR3*—both gene families that are expressed in the main olfactory epithelium—was recently reported for the greater sac-winged bat (*Saccopteryx bilineata*), a bat with no vomeronasal organ but with large-scented glands embedded in the wing membrane (Santos et al. 2016). As odorant-binding ligands have been identified for none of the hundreds of bat olfactory receptors (Hayden et al. 2014), some may respond to pheromonal cues.

Numerous neurobiological and behavioral studies have described strong interactions between the main olfactory epithelium and vomeronasal organ in detecting and discriminating pheromonal cues and initiating the behavioral response (Fraser and Shah 2014). Further investigation of the molecular mechanisms of the main olfactory system in bats may reveal redundancy amongst the two chemosensory system or unveil ways in which the main olfactory system compensates for vomeronasal loss in bats.

## Materials and Methods

RNA-seq libraries of the vomeronasal organ were generated for nine phyllostomid species (supplementary table S1, Supplementary Material online). Reads underwent quality control (supplementary table S2, Supplementary Material online), and were assembled using Oyster River Protocol v. 2.1.0 (MacManes 2018), which combines three assembly methods and quantitatively evaluates contig assembly based on several metrics. Vomeronasal tissue was validated by identifying the tissue-specific ion channel *Trpc2* β isoform transcripts (GenBank: MH010883-MH010888). Vomeronasal receptors were identified in the 9 new bat transcriptomes, and 16 published genomes (14 bats including the vampire bat, and the horse and dog) through a modified pipeline (Hayden et al. 2010) that implements a hidden Markov model algorithm to search for similar sequences using HMMER v. 3.12b trained from *V1r* sequence motif profiles (Mistry et al. 2013). Sequences were aligned for intact receptors only, and then for intact and pseudogenized receptors. The best-fit model of evolution was estimated for both alignments using ModelOMatic v. 1.01 (Whelan et al. 2015), and maximum likelihood gene trees were inferred from each alignment. Orthogroups were determined using the program UPhO (Ballesteros and Hormiga 2016). Rates of molecular evolution (*ω*) were estimated for bat and non-bat branch classes using Clade Model C in PAML v. 4.8 (Yang 2007) and RELAX (Wertheim et al. 2015).

## Supplementary Material

Supplementary data are available at *Genome Biology and Evolution* online.

## Supplementary Material

evz179_Supplementary_Data

## Notes

**Data deposition:** This project has been deposited to NCBI as a BioProject under the accession PRJNA563501 and to Dryad under the doi:10.5061/dryad.qr5s6b8. The characterized V1R and Trpc2 sequences have been deposited to Genbank under the accession MH010883-MH010888, MN432881-MN432889, MN461219-MN461225, and MG812189-MG812214. The raw RNA-seq reads were deposited to the NCBI Sequence Read Archive under the accession SRX6793373-SRX6793381.
