# Supplementary material for: Expressed Vomeronasal Type-1 Receptors (V1rs) in Bats Uncover Conserved Sequences Underlying Social Chemical Signaling
Source: Genome Biol Evol. 2019 Aug 19;11(10):2741–9. doi: 10.1093/gbe/evz179 (PMC6777432; doi:10.1093/gbe/evz179)
Supplement: evz179_Supplementary_Data [file evz179_supplementary_data.zip › v1r_manuscript_revision1_responses.pdf]

Associate Editor Recommendation:
Associate Editor: Chang, Belinda
Comments to the Author:
This is an interesting manuscript on a fascinating subject, but there are several major criticisms which need to be addressed in a substantially revised manuscript. Please pay careful attention to reviewers' comments and suggestions, particularly with respect to making the conclusions more carefully qualified and balanced. Please be sure to include a point-by-point summary of your revisions in response to reviewer comments.
Thank you for the consideration of this manuscript. We have tried to address the reviewer comments carefully and hope our responses suffice any concerns.

Reviewers' Comments:
Referee: 1
Comments to the Author
This paper deals with interesting aspects of the evolution of vomeronasal type-1 receptors in bats, a pertinent topic of research in these less studied taxa. While I found the retrieved results interesting, I think the experimental design could have been somewhat more elaborated and clearly the organization of the paper needs improvement overall. Conclusions are also speculative and unbalanced given the obtained results.
We appreciate your interest and have addressed your comments to the best of our abilities.

Below are some comments to take in consideration:
Pag. 5.
1) The authors showed the presence of TRPC2 and V1R. However, figure 1 has TRPC2 intact and pseudogenes in some species but for others such information is missing.
Thank you for pointing this out. TRPC2 had been sequenced and intact reading frames were identified in a few previous studies. To complete the missing data, for the remaining species in which TRPC2 had not yet been characterized but were included in our table (n=9), we performed the same BLASTn search that we did for the transcriptomes and aligned the identified gene to the TRPC2 ß isoform and identified whether the reading frame was intact. The identified BLAST sequences and alignments to the mouse gene will be deposited into Dryad. These changes are also now reflected in Figure 1.

NOTE: we removed the vicugna from the table because the species of the genome listed in the paper no longer corresponded to the species listed in Young, et al. 2010.

2) We can see that species with intact TRPC2 present intact V1Rs. However, for TRPC2 pseudogenes, in some cases there is intact V1R (ex. *Megaderma lyra*) but in others it was not found intact genes (ex. *Myotis lucifugus*). How do the authors explain this scenario?

In addition to the branch-site selection analysis comparing omega of bats and non-bats, we ran a selection analysis that included a third branch class, such that the comparison was non-bats, bats with intact *Trpc2*, and bats with nonfunctional *Trpc2*. While the likelihood ratio test was not significant, the estimate of omega is indeed higher for bats with a nonfunctional Trpc2. Our interpretation is that, these bats have one or two intact copies of V1rs, but the increased omega suggests that these genes may be evolving neutrally and have not yet accumulated a pseudogenizing mutation. Like humans, who lack an intact Trpc2, some animals still retain a low number of *V1r* copies that are still intact in the genome. This information is included in the supplement.

Pag. 9

3) In 2006, it was already reported that pheromones are detected by VR but VRs can detect other odorants and pheromones can also be detected by other systems, e.g. the olfactory system.

Baxi KN, Dorries KM, Eisthen HL. 2006. Is the vomeronasal system really specialized for detecting pheromones? Trends Neurosci. 29:1—7

Thank you for reminding us of this paper. We cleaned up the language in this section to reflect that this is an ongoing finding, rather than a novel idea that emerged from this paper.

Pag. 10
4) If the olfactory epithelium has capacity to maintain behaviors critical to survival that can hypothetically allow vomerolfaction relaxion, how do you support that bat vomerolfaction is redundant and frequently lost, whereas in other species we have lots of VRs duplications? Can environmental aspect or the upgrade of other chemosensorial system modulate these lost in bats?
We reworded this last paragraph to be more open-ended rather than conclusive, merely suggesting that the main olfactory system has the potential for maintaining pheromone signaling cues. If we were to speculate what is going on, if the reviewer is curious, is that bats are under extreme craniofacial adaptive selection in relation to their diet. It, perhaps, may not be that its not that selection has “relaxed” on bat vomerolfaction, but rather selection is stronger for extreme craniofacial adaptations and there is just not enough room in the skull for two chemosensory systems if one is sufficient. However, we do not currently have strong enough evidence to state this as a conclusion in the paper but hope to explore this point further in the future.

Referee: 2
Comments to the Author
Though linking specific chemosensory receptors with behavior is difficult, especially in non-model species, an evolutionary approach could help elucidate their functional roles. The manuscript by Yohe et al. aims to define a functional role for these receptors by identifying intact vomeronasal type 1 receptor transcripts from vomeronasal organs in various bat species via RNA sequencing analysis. De novo transcriptome assembly from RNA sequencing of bat vomeronasal organs was employed to define intact V1rs. The authors illustrate highly-variable patterns of copy number and intact v. pseudogene counts among bat lineages. Interestingly, some receptors were found to be highly orthologous to the horse and dog outgroups. Overall, the authors' careful work is likely to move our understanding of V1R evolution forward in a significant way. I have several points for the authors to consider to improve the manuscript.
Thank you for the supportive comments. We have tried to address your comments to the best of our abilities.

1. Title: this reviewer does not agree that the current work "uncover conserved mechanisms of social chemical signaling". Function cannot be supported from simply receptor sequence.
We have changed the title to the following: “Expressed *vomeronasal type-1 receptors* (*V1rs*) in bats uncover conserved sequences underlying social chemical signaling”

2. Unclear how important any behavior linked to VNO is to bats/laurasiatheria if numerous species lack intact V1Rs, TRPC2, or AOB.
While our previous work supports the independent degradation of trpc2 across many bat lineages, these losses are unusual with respect to other mammals. In effect, the norm is for mammals in general and laurasiatherians in particular to conserve the entire system, including molecular machinery and organs. Outside bats, the exceptions in laurasiatheria tend to be lineages with obvious constraints on chemosensation, such as whales and seals. Therefore the numerous species in which the system breaks down are still exceptional and remain unexplained only among bats. We clarify its

relevance and now note this on p. 1 par. 2, L61.

3. Trinity *de novo* assembly has seen 4 major updates from the version used in the manuscript and may improve results such as the removal of the duplicate receptor if the most current version is used.

Thank you for bringing this to our attention, as after a bit of exploration, we noticed that both different assembly versions and assembly methods resulted in differences in numbers of intact receptors identified (ranging from 1-5 fewer or less than previously identified. Determining the resolution to this issue is the main reason for the delay in this manuscript. Our approach now implements the Oyster River Protocol (MacManes 2018), a transcriptome assembly pipeline that combines multiple assembly approaches, quantitatively evaluates the quality of the assembly, and pools unique high-quality contigs from different assemblies. Briefly, the protocol performs the following analyses: (1) additional trimming and error correction; (2) assembly using Trinity v. 2.8.4 (Grabherr et al. 2011), Trans-Abyss v. 2.0.1, and SPAdes v. 3.13.0; (3) merging of assemblies via OrthoFinder v. 2.2.6 (Emms & Kelly 2015); and (4) assembly evaluation using TransRate v. 1.0.2 (Smith-Unna et al. 2016) and BUSCO v. 3.0.1 (Waterhouse et al. 2017). We now have high confidence in the resulting assemblies and receptors identified. We are in the process of updating sequences for GenBank.

4. Tests for selection are not extensive. In addition to omega, branch-site tests are necessary for such conclusions. Also, the authors should make it clear about which hypotheses would be accepted or rejected, given the findings of purifying and/or neutral selection.

Even in our original version, we ran Clade Model C within PAML and RELAX within Hy-Phy, both of which are branch-site approaches. We repeated the above selection with the new alignments that included the new receptors detected with the new assembly approaches. Table 1 and 2 demonstrate branch-site results, showing omega estimates for the different branch classes, as well as the three site classes. We also explicitly state these results in terms of our hypothesis.

5. On page 8, the authors have a discussion of site-specific behaviors, but it is unclear as to whether they are discussing DNA sites or aa sites.

We are referring to codon sites and have reworded to remedy this. Anytime “site” is mentioned, we have clarified to read “codon sites”.

6. Regarding coevolution of Trpc2, the VNO, and intact V1Rs, their comparisons are suggestive of this pattern, but they should be more circumspect in their language given that their taxon sampling is pretty limited, and the patterns in Sorex and Erinaceus (outgroups) and Pternotus (bat) don’t really follow the pattern.

We have made our language more precise regarding the pattern. To clarify, while there are exceptions (e.g., *Megaderma*, *Rhinolophus*, and *Hipposideros* each have one putatively functional V1R despite lacking the VNO and having a disrupted trpc2), the pattern holds for the most part. First, *Pteronotus parnellii* has every component of the vomeronasal system. Second, the other laurasiatherian outgroups have an intact Trpc2 from our blast search (see updated Figure 1 and supplement). Finally, we have shown in a previous analysis (Yohe & Davalos (2018) in *BJLS*) that quantitatively demonstrated a tight coupling of selection pressures on the genetic and morphological components of the system.

7. Discussion on the discrepancy between the detected V1R genes for horse and dog genomes from Young et al. would be helpful for assessing method: was a different genome version actually used? Selection/training set differences? What sequences were detected by Young but not here?

We noted the differences amongst our two data sets and explained it was likely due to both different versions of genomes available and different search algorithms for V1r genes (HMMR v. BLAST).

L142: “We also detected differences in receptors (between 3-6 genes) from the horse and dog genomes than had been previously reported (Young et al. 2010). Differences are likely due to the availability newer versions of the dog and horse genomes and our use of hidden Markov models rather than BLAST to characterize *V1r*s. We emphasize, however, that the reported number of *V1r* genes per species should be considered a dynamic value and may change as genome assemblies and annotation methods improve.”

8. The word “Darwinian selection” is the community standard to describe “positive selection.”
Addressed where appropriate.

9. Alignment of the V1Rs Running MAFFT e-ins-i could be a more appropriate accuracy oriented alignment algorithm for divergent membrane spanning proteins like GPCRs than FFT-NS-1.
Thank you for the suggestion. We reran the alignment that included psuedogenes with the e-ins-I algorithm and noticed slight improvements. Translation alignment was still used for the intact genes.

10. The authors should cite the Isogai et al. (2011) paper arguing for a possible heterospecific function for the intact V1Rs.
We have cited this paper on page 9 during our discussion of alternative roles of V1rs.

11. Line 147 word missing – orthogroups are “ ” to be evolving
We adjusted this to “orthogroups may be evolving”.

12. Line 79 word missing - conserved “a” functional Trpc2
Addressed.

13. Line 67 word misspelled – “genearates”
Addressed.
