# Supplementary material for: Expressed Vomeronasal Type-1 Receptors (V1rs) in Bats Uncover Conserved Sequences Underlying Social Chemical Signaling
Source: Genome Biol Evol. 2019 Aug 19;11(10):2741–9. doi: 10.1093/gbe/evz179 (PMC6777432; doi:10.1093/gbe/evz179)

## Supplemental Methods

*Tissue collection*: RNA-seq of the vomeronasal organ required freshly collected specimens. We targeted nine species of divergent phyllostomids, all hypothesized to have a functional vomeronasal organ based on a previous study that indicated an intact *Trpc2* open reading frame (Yohe et al. 2017). One individual per species was collected. Sampling took place at five different field sites over the course of three expeditions, one in Belize, one in Peru, and one in Costa Rica (Supplementary Table S1 describes the nine species and locality information). All specimens were collected, handled, and euthanized in accordance with Stony Brook University IACUC permit 323555-2 for Belize, 614763-3 for Peru, and 448712-3 for Costa Rica. Bats were euthanized using an excess of isofluorane, and the maxillary rostrum was immediately dissected and placed in vials of RNAlater. Tissues were incubated overnight at ~4ºC to allow the RNAlater to completely permeate the dense tissue, and then flash-frozen in liquid nitrogen. Protocols for initial dissection and tissue preservation are found in Yohe et al. (2019). Vomeronasal organs was carefully dissected from the maxilla on a sterile cold table under a microscope, following a published video dissection protocol (Brechbühl et al. 2011). RNA was immediately extracted from the vomeronasal organ.

*RNA extractions and sequencing*: We extracted total RNA from the vomeronasal organ from nine species using the Qiagen RNeasy Micro Kit (Product ID: 74004). Vomeronasal organ tissue was placed in a glass mortar and ground with a pestle until all tissue was disrupted. Grinding with the pestle took place for ~5 minutes with enough force to create a vortex that was sufficient to homogenize the cells. We followed the protocol for the "Purification of Total RNA from Animal and Human Tissues" (Qiagen), with the following modifications. Tissue was not flash frozen after dissection since it was recently thawed (but kept cold) for the dissections. All tissue grinding took place on a sterile cold table kept cold on dry ice. We also added the suggested volume of carrier RNA to facilitate the extraction of small quantities of RNA found in neural tissues, with the understanding that this may be present in downstream analyses. We also incubated the samples with the DNase treatment for 8 minutes, as opposed to the suggested 15 minutes to avoid degradation of RNA. The RNA quantification and quality assessment were performed with the Agilent 2100 Bioanalyzer by BGI, who also prepared and sequenced the cDNA libraries from the total RNA extractions using the Illumina HiSeq™ 4000 platform. Each sample resulted in about 6 gigabases of 100-150 base-pair (bp) paired-end reads.

*Quality control and transcriptome assembly:* Due to the duplicative nature of *V1r* genes, careful consideration was given when designing the pipeline for read quality control and read assembly. We first used several scripts found within the BBTools bioinformatics package (https://sourceforge.net/projects/bbmap/) for read processing and filtering. Low quality reads less than 25 bp (minlen=25) were discarded using the bbduk.sh script. Reads were trimmed from both ends (qtrim=rl) until the average read quality was 10 or greater (trimq=10), or otherwise the low-quality read was removed. To account for variation in assembly approaches, cleaned reads were submitted to the Oyster River Protocol pipeline (MacManes 2018) that performs several assembly programs, evaluates the quality of each assembly, and pools high quality contigs across different

assembly approaches. Details of this transcriptome assembly protocol and performance for large gene families such as chemosensory receptors are available in Yohe, Davies, et al. (2019).

*Tissue validation*: Dissection of the vomeronasal organ is challenging. Therefore, to ensure we had sequenced vomeronasal epithelial tissue, we checked for the expression of the *Trpc2* ß-isoform, a primary ion channel in vomeronasal sensory neuron signal transduction. This isoform is mainly expressed in the vomeronasal organ (Mast et al. 2010), and the presence of *Trpc2* transcripts indicates a high probability of vomeronasal cell types. Using previously published *Trpc2* exon sequences (Yohe et al. 2017), we queried for its expression in the bat vomeronasal transcriptomes using tblastn and a 1e-06 E-value threshold per transcriptome. We also wanted to ensure that the *V1r*s in our transcriptome were a thorough representation of intact vomeronasal receptors. Thus, we compared the *V1r*s from the *Desmodus rotundus* transcriptome to those of the genome (Zepeda Mendoza et al. 2018). tblastn searches of *Trpc2* were also performed for genomes in which the *Trpc2* sequence had not yet been characterized.

*V1r identification*: *V1r* genes are approximately 900-bp, single-exon sequences (Dulac & Axel 1995). Although *V1r* genes are highly diverse, their duplicative nature and conserved G-protein coupled receptor structure allow for confident identification. To search for *V1r*s in the bat transcriptomes and available genomes, we built sequence motif profiles that trained the hidden Markov model algorithm to search for similar sequences using HMMER v. 3.12b (Eddy 2010). As the diversity of receptors in the bat transcriptomes was unknown, we trained the program with extensive mammalian sequence diversity. We downloaded 1,844 annotated, intact *V1r* sequences from across mammals from GenBank, and designed HMMER profiles from an alignment built using transAlign (Bininda-Emonds 2005). The alignment of the trainer sequences is available from Dryad. For the transcriptomes, we incorporated this HMMER profile into the Olfactory Receptor Assigner (ORA) v. 1.9.1(Hayden et al. 2010). This is a Bioperl (v. 1.006924) program originally designed to characterize olfactory receptors and identify pseudogenes among contigs using HMMER, and we extended this program to also search for *V1r* genes. We searched for *V1r* sequences in all available bat genomes (14 species, including two noctilionoids [*Pteronotus parnellii* and *Desmodus rotundus*] and one miniopterid [*Miniopterus natalensis*]), as well as in the outgroup species of the horse and dog, which are two related laurasiatherians with well-annotated genomes. Sequences resulting from the HMMER analyses shorter than 650 bp were not included in the analyses, as sequences shorter than this cannot encode a proper seven potential transmembrane domain (Hayden et al. 2010). All sequences were run through the ORA pipeline to identify open reading frames and pseudogenes. For genomic sequences, a gene was considered a pseudogene if it had a frameshift mutation or if a stop codon that truncated the sequence to less than 800 bp. As a short transcript does not necessarily imply a short gene, but instead a fragment in the transcriptomes, a gene was considered functional if it was longer than 600 bp and had no stop codon or frameshift mutation. To identify *V1r* genes from available genomes, we used "nhmmer" within the HMMER program to compare sequences with significant homology to the sequences trained in the alignment. We searched for *V1r* sequences in all available bat genomes including *Desmodus rotundus* (Zepeda Mendoza et al. 2018), *Eidolon helvum*

(GCA_000465285.1), *Eptesicus fuscus* (GCF_000308155.1), *Hipposideros armiger* (GCA_001890085.1), *Megaderma lyra* (GCA_000465345.1), *Miniopterus natalensis* (GCA_001595765.1), *Myotis brandtii* (GCA_000412655.1), *Myotis davidii* (GCA_000327345.1), *Myotis lucifugus* (GCA_000147115.2), *Pteronotus parnellii* (GCA_000465405.1), *Pteropus alecto* (GCA_000325575.1), *Pteropus vampyrus* (GCA_000151845.2), *Rhinolophus ferrumequinum* (GCA_000465495.1), *Rhinolophus sinicus* (GCA_001888835.1), and *Rousettus aegyptiacus* (GCA_001466805.2), as well as two related laurasiatherians: *Equus caballus* (GCA_000002305.1), and *Canis familiaris* (GCA_000002285.2). Identified sequences were pulled out of the genome with "easel" post-processing tools (Eddy & Wheeler 2010).

*Sequence alignment*: A *V1r* sequence of the zebrafish *Danio rerio* (DQ8876141), which has been used as an outgroup in previous analyses of mammalian *V1r*s (Young et al. 2010), was included in each alignment to root the tree. We first aligned sequences with intact and sufficiently long reading frames using translation alignment within Geneious v. 8.1.7 (Bininda-Emonds 2005; Kearse et al. 2012). The alignment included all intact receptors from bats, dog, and horse. We then inferred a second alignment with all intact *V1r*s and bat *V1r* pseudogenes using MAFFT v. 7.017 with the e-ins-i algorithm, 200PAM/k=2 scoring matrix, gap open penalty of 1.83, and an offset value of 0.123 (Katoh & Standley 2013).

*Gene tree inference*: Two tree inference approaches were undertaken for both the alignment with intact genes and the alignment that contained both pseudogene sequences and intact genes. With the alignment of intact *V1R*s, we tested for the best-fit model of evolution and inferred gene trees from the alignments using maximum likelihood. With the alignment of intact *V1r*s, we tested for the best-fit model of evolution using ModelOMatic v. 1.01 (Whelan et al. 2015), a program that compares nucleotide, codon, and protein models of evolution. We removed any stop codons at the ends of genes or transcripts. The gene tree was estimated with this same alignment using Garli v. 2.0.167 (Zwickl 2006) on the CIPRES Science Gateway server (Miller et al. 2010), as it implements codon models. We performed this inference with 8 search replicates to ensure convergence on a similar tree solution and performed 1000 bootstrap replicates to estimate branch support. Bootstrap trees were summarized using SumTrees v. 4.0.0 within Dendropy v. 4.0.3 (Sukumaran & Holder 2010). For the total gene alignment, the reading frame was no longer easily detectable, and we used used the GTRCAT model of evolution implemented in maximum likelihood program RAxML v. 7.2.8 (Stamatakis 2006), using 1,000 bootstrap replicates for branch support.

*Ortholog identification*: Like most chemosensory genes, *V1r*s frequently duplicate and lose function, making it a challenge to distinguish orthologous genes from paralogs. In order to measure the strength of selection in orthologous genes across different species, we characterized the orthologous gene groups (orthogroups) of *V1r*s using the orthology assessment program UPhO (Ballesteros & Hormiga 2016). This program uses an unrooted approach to identify genes that form a monophyletic group across different species. UPhO prunes the gene tree into orthogroups, while also allowing in-paralogs, or genes within an orthogroup that have duplicated since a species

diverged, to remain in the tree. While it was originally intended to assess orthology for phylogenomic inference, the program is also useful to identify orthologous subfamilies within a large gene family (Dunn & Munro 2016). We identified orthogroups and all in-paralogs from an unrooted gene tree, setting the minimum number of taxa per orthogroup to five species.

*Selection analyses*: We tested whether the intact *V1r*s in bats were evolving at a different rate than the functional receptors of closely related laurasiatherian outgroups. To characterize the mode and strength of selection acting on the two groups, we quantified the ratio of rates of nonsynonymous to synonymous substitutions ($dN/dS$, also known as $\omega$) for the entire gene tree of intact *V1rs*, as well as for individual *V1r* orthogroups. We used the gene tree inferred from the codon sequence alignment to run a clade model using the codeml package of PAML v. 4.8b (Yang 2007). This model estimates ω for each specified branch class, and also categorizes each codon site into three different rate classes. These rate classes indicate sites evolving under purifying selection ($\omega_1$), neutral evolution ($\omega_2$), and a third class that is free to vary from a different category than the first two ($\omega_3$). We compared a null model (M2a_rel), in which the same three classes of $\omega$ was estimated for all branches on the tree ($\omega_{\mathrm{background}}$), against an alternative model with two distinct clade classes (Clade Model C), in which the background branch rate ($\omega_{\mathrm{background}}$) included all intact *V1r*s for the horse and dog, and a foreground branch class ($\omega_{\mathrm{bats}}$) that includes the branches for all bat *V1r* genes. We also ran the Clade Model C analysis for each orthogroup that had more than five species and at least six genes within the orthogroup tree and had both test groups present in the orthogroup. To test whether the two-clade class model was preferred over the null, we performed likelihood ratio tests.

We also used RELAX to help distinguish between relaxed and positive selection for the two branch classes(Wertheim et al. 2014). RELAX uses a branch-site random effects likelihood model to estimate three site classes of $\omega$ for each branch on the tree. It then calculates a parameter $k$, which optimizes the strength of selection and transforms $\omega$ estimates by raising them to a power of $k$. When $k > 1$, high and low branch classes are pushed away from 1, suggesting an intensification of selection. When $k < 1$, the high and low branch class $\omega$ move closer to 1, suggesting relaxed selection. We performed this analysis for the entire gene tree.

## Supplemental Results

*RNA-seq*: All extracted RNA resulted in quantity and quality sufficient for sequencing, in which the RNA Integrity Number (RIN) was greater than 8.0 (Table S1). Resulting read counts after quality control and processing ranged from ~36 million to 64 million bp. Most assemblies resulted in high quality transRate scores (Table S2). One exception was the three *Carollia* species, which may explain low recovery number of *V1r* transcripts. While these extractions had a high RIN value (Table S1), overall RNA quantity was low. In addition to standard assembling and mapping error, the trimming of chimeric contigs may have affected the failure of some reads to map.

*Tissue validation*: Transcripts of the *Trpc2* β isoform (~2,600 bp) were identified in the transcriptome of each bat species. For three species, the entire *Trpc2* coding sequence was recovered. In the remaining six species, the following base pair coverage of transcript fragments

for *Trpc2* was recovered: *A. fraterculus* (815 bp), *C. brevicauda* (363 bp), *C. perspicillata* (736 bp), *C. sowelli* (526 bp), *C. castanea* (825 bp), *and S. lilium* (356 bp). These sequences have been deposited in GenBank (MH010883-MH010888).

*Sequence alignment*: For the intact *V1r* alignment, the alignment contained 98 coding sequences, spanned 362 codon positions, and had 55.0% pairwise identity, with the average sequence length spanning 293 ± 24.6 codons. The best-fit model of evolution was a Codon Reduced F1X4 with a *dN*/*dS* parameter varying across four rate categories. Figure 3 depicts the resulting tree. All eight search replicates from the maximum likelihood inference converged on a similar solution. For the alignment that included both intact receptors and pseudogenes, the aligned sequences spanned 1,628 base pairs for 278 genes. Some of these sites included gaps from insertions of some pseudogenes. Figure 2 depicts the resulting tree.

*Orthology inference:* Twenty-seven orthogroups were recovered using UPhO. Seven of these orthogroups contained only a single gene and most contained only two or three genes. Thus, our analysis recovered a total of three orthogroups with more than five species (Fig. 2, orthogroup A–C) and only two with enough sequences to be used in the selection analyses (Fig. 2, orthogroup A,C). There were no orthogroups with more than six genes that solely contained bats, suggesting all bats share orthologs with either the horse or dog lineages. Finding these first bat *V1r* intact open reading frames both enables comparative analyses with other mammals and suggests conserved function.

*Selection analyses*: We compared the ratio of rates of nonsynonymous to synonymous substitutions for bats to that of a background rate of ancestral genes from the horse and dog. When considering rates for the entire tree of intact *V1r*s, the null model of the ω class estimates for the whole tree (i.e. not multiple clades) was not rejected by the likelihood ratio test (Table 1; $\chi^2_{(1)} = 2.1$ $P = 0.15$), finding no statistically significant difference between rates in bats and rates of the background branches. Results from RELAX corroborate those of PAML (Table 2). The null model could not be rejected ($\chi^2_{(1)} = 0.65$ $P = 0.42$), there was neither a difference in $\omega$ across branch classes, nor transformation of $\omega$ values ($k$ =1).

**Supplemental References**

Ballesteros JA, Hormiga G. 2016. A new orthology assessment method for phylogenomic data: Unrooted phylogenetic orthology. Mol. Biol. Evol. 33:2117–2134. doi: 10.1093/molbev/msw069.

Bininda-Emonds ORP. 2005. transAlign: using amino acids to facilitate the multiple alignment of protein-coding DNA sequences. BMC Bioinformatics. 6:156. doi: 10.1186/1471-2105-6-156.

Brechbühl J, Luyet G, Moine F, Rodriguez I, Broillet M-C. 2011. Imaging pheromone sensing in a mouse vomeronasal acute tissue slice preparation. J. Vis. Exp. e3311. doi: doi:10.3791/3311.

Dulac C, Axel R. 1995. A novel family of genes encoding putative pheromone receptors in mammals. Cell. 83:195–206. doi: 0092-8674(95)90161-2.

Dunn CW, Munro C. 2016. Comparative genomics and the diversity of life. Zool. Scr. 45:5–13. doi: 10.1111/zsc.12211.

Eddy S. 2010. HMMER3: a new generation of sequence homology search software. http://hmmer.janelia.org.

Eddy S, Wheeler TJ. 2010. HMMER user's guide version 3.0. Dep. Math. Washingt. Univ. St. Louis, MO.

Hayden S et al. 2010. Ecological adaptation determines functional mammalian olfactory subgenomes. Genome Res. 20:1–9. doi: 10.1101/gr.099416.109.

Katoh K, Standley DM. 2013. MAFFT multiple sequence alignment software version 7: improvements in performance and usability. Mol. Biol. Evol. 30:772–780.

Kearse M et al. 2012. Geneious Basic: an integrated and extendable desktop software platform for the organization and analysis of sequence data. Bioinformatics. 28:1647–1649.

MacManes MD. 2018. The Oyster River Protocol: a multi-assembler and kmer approach for *de novo* transcriptome assembly. PeerJ. 6:e5428. doi: 10.1109/ECTC.2009.5074081.

Mast TG, Brann JH, Fadool DA. 2010. The TRPC2 channel forms protein-protein interactions with Homer and RTP in the rat vomeronasal organ. BMC Neurosci. 11:1–16. doi: 10.1186/1471-2202-11-61.

Miller M a., Pfeiffer W, Schwartz T. 2010. Creating the CIPRES Science Gateway for inference of large phylogenetic trees. In: Procedings of the Gateway Computing Environments Workshop (GCE). Ieee: New Orleans pp. 1–8. doi: 10.1109/GCE.2010.5676129.

Stamatakis A. 2006. RAxML-VI-HPC: maximum likelihood-based phylogenetic analyses with thousands of taxa and mixed models. Bioinformatics. 22:2688–90. doi: 10.1093/bioinformatics/btl446.

Sukumaran J, Holder MT. 2010. DendroPy: a Python library for phylogenetic computing. Bioinformatics. 26:1569–1571.

Wertheim JO, Murrell B, Smith MD, Kosakovsky Pond SL, Scheffler K. 2014. RELAX: Detecting relaxed selection in a phylogenetic framework. Mol. Biol. Evol. 32:820–832. doi: 10.1093/molbev/msu400.

Whelan S, Allen JE, Blackburne BP, Talavera D. 2015. ModelOMatic: Fast and automated model selection between RY, nucleotide, amino acid, and codon substitution models. Syst. Biol. 64:42–55. doi: 10.1093/sysbio/syu062.

Yang Z. 2007. PAML 4: phylogenetic analysis by maximum likelihood. Mol. Biol. Evol. 24:1586–91. doi: 10.1093/molbev/msm088.

Yohe LR, Davies KT, et al. 2019. Targeted sequence capture outperforms RNA-Seq and degenerate-primer PCR cloning for sequencing the largest mammalian multi-gene family. bioRxiv. 1–34. doi: 10.1111/j.1478-5153.2006.00182_4.x.

Yohe LR, Devanna P, et al. 2019. Tissue collection of bats for -*omics* analyses and primary cell culture. J. Vis. Exp. JoVE. In Press.

Yohe LR et al. 2017. *Trpc2* pseudogenization dynamics in bats reveal ancestral vomeronasal signaling, then pervasive loss. Evolution (N. Y). 71:923–935. doi: 10.1111/evo.13187.

Young JM, Massa HF, Hsu L, Trask BJ. 2010. Extreme variability among mammalian *V1R* gene families. Genome Res. 20:10–18. doi: 10.1101/gr.098913.109.

Zepeda Mendoza M et al. 2018. Hologenomic adaptations underlying the evolution of sanguivory in the common vampire bat. Nat. Ecol. Evol. 2:659–668. doi: 10.1038/s41559-018-0476-8.

Zwickl DJ. 2006. Genetic algorithm approaches for the phylogenetic analysis of large biological sequence datasets under the maximum likelihood criterion. Ph.D. Diss. Univ. Texas Austin.

**Table S1**. Species and specimen information sampled in this study. RIN is the RNA integrity number, and the minimum standard for most protocols, including those performed at the Beijing Genome Institute, is 8.0.

| Species | Field # | Locality | Sex | [RNA] (ng/µL) | RIN |
|---|---|---|---|---|---|
| *Artibeus fraterculus* | PE005 | Suyo, Peru | M | 76 | 8.5 |
| *Sturnira ludovici* | PE019 | Faique, Peru | M | 53 | 8.9 |
| *Sturnira lilium* | NBS1146 | Lamanai, Belize | M | 50 | 8.7 |
| *Carollia brevicauda* | PE111 | Loreto, Peru | M | 152 | 8.6 |
| *Carollia perspicillata* | LS070 | La Selva, Costa Rica | M | 138 | 7.9 |
| *Carollia sowelli* | LS073 | La Selva, Costa Rica | M | 132 | 8.4 |
| *Carollia castanea* | LS084 | La Selva, Costa Rica | M | 80 | 8.2 |
| *Glossophaga soricina* | PE022 | Faique, Peru | F | 70 | 8.4 |
| *Desmodus rotundus* | NBS1170 | Lamanai, Belize | M | 62 | 9.1 |

**Table S2**. Metrics and results from RNA-seq and assemblies for the vomeronasal organ transcriptomes. Total PE reads are the number of paired end reads used in the Trinity assembly, after quality control and processing. Assembled contigs are resulting assembled transcripts after chimeric transcripts were removed. Reads were mapped back to the assembled transcriptome using Bowtie2 v. 2.3.0 under the default global alignment.

| **Species** | **TransRate Score** | **TransRate Optimal** | **BUSCO score n: 303** |
|---|---|---|---|
| *Artibeus fraterculus* | 0.510 | 0.566 | C:96.0% [S:53.8%, D:42.2%] F:4.0%, M:0.0% |
| *Sturnira ludovici* | 0.461 | 0.583 | C:98.0% [S:50.5%, D:47.5%] F:2.0%, M:0.0% |
| *Sturnira lilium* | 0.591 | 0.633 | C:97.3% [S:80.5%, D:16.8%] F:2.6%, M:0.1% |
| *Carollia brevicauda* | 0.514 | 0.580 | C:97.4% [S:49.2%, D:48.2%] F:2.3%, M:0.3% |
| *Carollia perspicillata* | 0.166 | 0.581 | C:98.7% [S:58.4%, D:40.3%] F:1.3%, M:0.0% |
| *Carollia sowelli* | 0.189 | 0.623 | C:97.4% [S:49.5%, D:47.9%] F:2.6%, M:0.0% |
| *Carollia castanea* | 0.176 | 0.626 | C:97.3% [S:48.8%, D:48.5%] F:2.6%, M:0.1% |
| *Glossophaga soricina* | 0.533 | 0.586 | C:97.7% [S:57.4%, D:40.3%] F:2.0%, M:0.3% |
| *Desmodus rotundus* | 0.596 | 0.632 | C:95.4% [S:69.0%, D:26.4%] F:4.0%, M:0.6% |

**Figure S1**. Unrooted gene tree depicting recovered intact *V1R*s from the transcriptome and genome of *Desmodus rotundus*. While there were some *V1R*s in the genome not present in the transcriptome, all *V1R*s from the transcriptome were present in the genome, with the exception of one duplicate receptor identified in the transcriptome. The alignment was performed with transAlign and the tree was inferred using FastTree in Geneious 8.1.7.

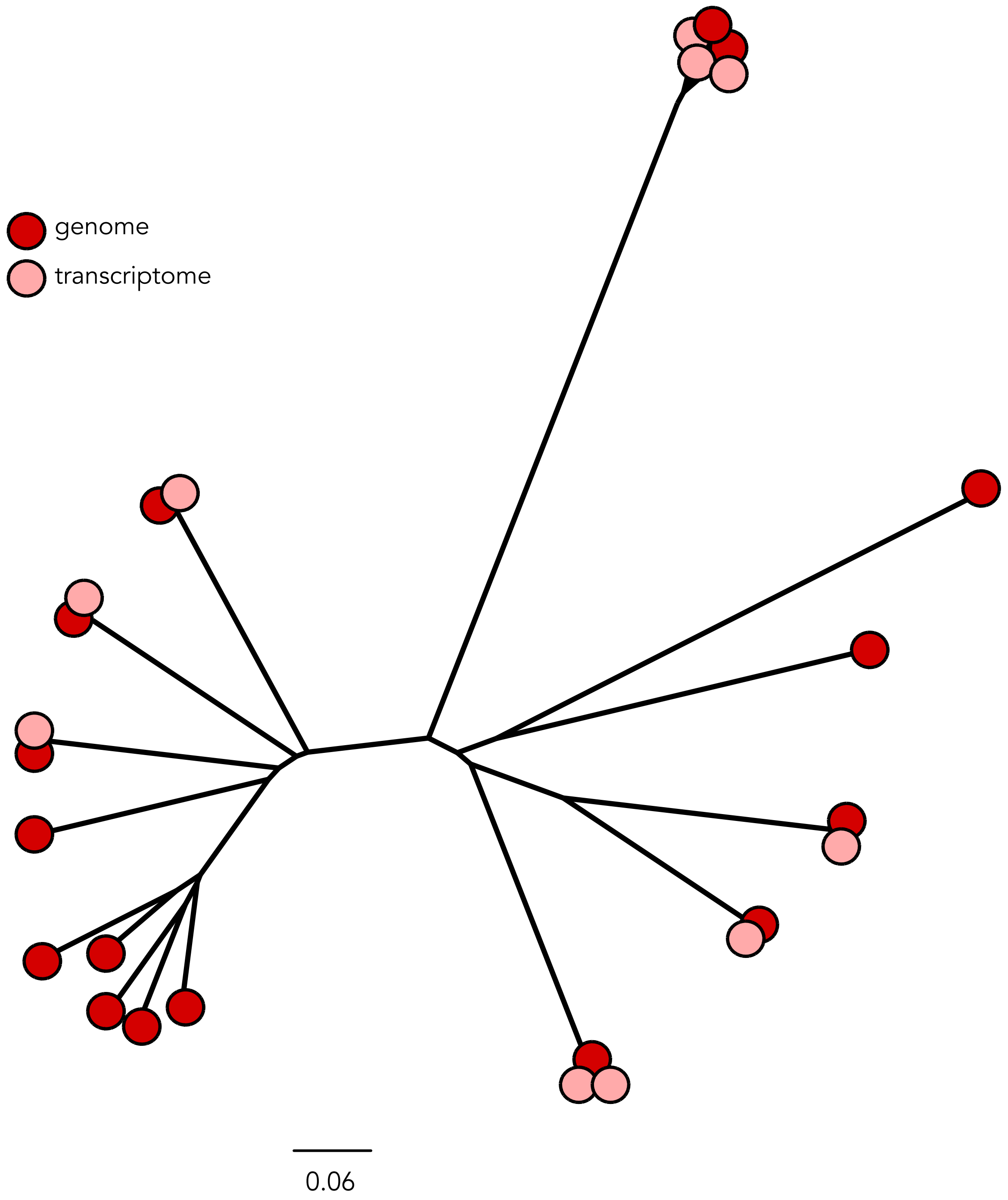

Supplement: evz179_Supplementary_Data [file evz179_supplementary_data.zip › V1R_manuscript_supplement_revision1_clean.pdf]
